# Segmentation of Structural Elements from 3D Point Cloud Using Spatial Dependencies for Sustainability Studies

**DOI:** 10.3390/s23041924

**Published:** 2023-02-08

**Authors:** Joram Ntiyakunze, Tomo Inoue

**Affiliations:** Department of Environmental and Heritage Design, Graduate School of Design, Kyushu University, 4-9-1 Shiobaru, Minami-ku, Fukuoka 815-8540, Japan

**Keywords:** point cloud, planar patches, segmentation, classification, occlusion, structural elements

## Abstract

The segmentation of point clouds obtained from existing buildings provides the ability to perform a detailed structural analysis and overall life-cycle assessment of buildings. The major challenge in dealing with existing buildings is the presence of diverse and large amounts of occluding objects, which limits the segmentation process. In this study, we use unsupervised methods that integrate knowledge about the structural forms of buildings and their spatial dependencies to segment points into common structural classes. We first develop a novelty approach of joining remotely disconnected patches that happened due to missing data from occluding objects using pairs of detected planar patches. Afterward, segmentation approaches are introduced to classify the pairs of refined planes into floor slabs, floor beams, walls, and columns. Finally, we test our approach using a large dataset with high levels of occlusions. We also compare our approach to recent segmentation methods. Compared to many other segmentation methods the study shows good results in segmenting structural elements by their constituent surfaces. Potential areas of improvement, particularly in segmenting walls and beam classes, are highlighted for further studies.

## 1. Introduction

The existing stock of buildings gives an opportunity to analyze and evaluate the sustainability performance of the building sector, which is regarded as one of the leading sources of carbon emissions [1]. Buildings annually consume about 48% of overall global energy, including operational and embodied energy [2]. Particularly, the structural systems of buildings contribute to most of the embodied energy due to the high demand for cement, which accounts for 5–7% of total global carbon emissions [3]. This necessitates up-to-date and reliable assessments of embodied carbon emissions and the lifecycle assessment (LCA) of buildings. In this case, point clouds provide a potent ability to identify and segment structural elements for sustainability studies. Current studies have put efforts to integrate LCA with the building information models (BIM) [4,5,6]; however, there are fewer buildings with updated as-built BIM [7].

Three-dimensional point clouds generated from laser scanning, photogrammetry, and video-grammetry have shown great potential due to their high-detail and accurate geometrical representation of building components [8,9]. In the built environment, the application of point clouds can be summarized as twofold [8]: the 3D model reconstruction and quality inspection of geometry properties. In both applications, the use of point clouds is commonly related to structural analysis, which essentially requires accurate detection and segmentation processes and often needs to integrate the prior knowledge of the object’s features and extract any relevant information from the point cloud. Extracting spatial information from the point cloud is essential to derive any meaningful linkage and relation among entities [10,11]. However, the critical challenge is that point cloud scenes are characterized by raw and aggregate datasets [10], which are time-consuming and prone to errors in the processing of the data [12]. In addition, the presence of clutter and occlusions in the scenes further complicates the segmentation process.

Many approaches involving the recognition and segmentation of building components from point clouds have relied on the detection of local geometric primitives, predominantly planes and cylinders; however, most of them have failed to explicitly identify and classify the structural components for further processing [12,13]. Moreover, most of these approaches have proven to be sensitive to noise and computationally expensive [14,15], especially when working with large point clouds [15]. Recent studies have incorporated the contextual information of structural elements in the segmentation pipeline [16,17,18,19]; however, their approaches have solely been illustrated in construction-related scenes where the building components are relatively more exposed and visible to sensors compared to those of existing and occupied buildings.

**Figure 1:**
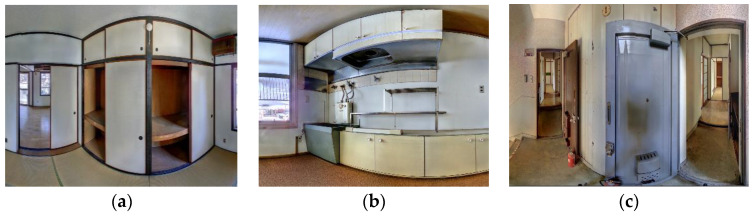
Various forms of occlusions are present in an occupied building: (**a**) large wardrobe concealing one face of the wall; (**b**) upper cabinet resembling a short-span beam; (**c**) door leaning and occluding a wall surface.

Existing buildings normally provide less exposure to permanent structures due to occlusions from fittings and fixtures present in the scenes. Some occlusions, for instance, furniture and false (suspended) ceilings, can exhibit dimensional sizes and/or orientations that are proportional and similar to those of generic building elements such as walls [13], slabs, and beams as illustrated in Figure 1. Furthermore, occlusions predispose point clouds to miss data, especially during the estimation of local saliency features [17], which can cause incorrect segmentations due to disconnected surface patches. 

As such, we propose a new approach that can robustly segment structural points from nearby non-structural objects while handling the problem of missing data. The approach introduces a selection of principal pairs of detected planar patches and spans their corresponding planes to locate and merge with the remotely disconnected patches from the same structural surfaces. The patch-pairing is used to assimilate the surface symmetry of common structural components in the segmentation pipeline.

Our approach can overcome various magnitudes of missing data and handles point clouds of different scales and sample distributions. The case study was conducted on a multistory building with planar surfaces; however, the proposed approach can be utilized in other concrete-based structures.

The main objective of this study is to propose a segmentation method that can retrieve fragmented and disconnected planar surfaces of the same structural elements due to extensive occlusions and unprecedented variations of point density. 

Other contributions of this paper include:

- It accelerates the rate of estimation of the local saliency features in a noisy and large point cloud. This is done by decomposing the point cloud into well-defined voxels for reliable statistical computations using an optimum number of samples in voxels.
- It develops novel techniques for classifying the detected planar surfaces into well-defined structural elements using the spatial dependency and topology of structural forms.

The remainder of this paper is organized as follows. Related works are described in Section 2. Methodology and experimental results are provided in Section 3 and Section 4, respectively. Discussions about the results obtained from the experiments are provided in Section 5, and in Section 6 we present the study conclusions. 

## 2. Related Works

### 2.1. Literature Search

Several pieces of research related to the segmentation and classification of point clouds in building and civil works have been conducted over the years, and to best utilize the recent and relevant literature, the authors mainly concentrated on searching for research papers published in a few reputable international journals and conferences. Related publications indexed under popular directories such as Web of Science, Scopus, and DOAJ were searched using a combination of keywords related to the processing and application of point clouds in construction and the built environment. Keywords used were *point cloud*, *laser scan*, *segmentation*, *classification*, *building*, *construction*, *existing building*, *structure*, *and infrastructure*. The supporting literature was sought using the same keyword search *in* Google Scholar, Scopus, and ResearchGate. A further search was also conducted from the background and method citations from the initial papers reviewed to gain more insights and focus on the subjects, which also included technical reports and theses.

### 2.2. Point Cloud Geometry

The reconstruction of 3D models from point clouds relies on the accurate recognition and segmentation of structural features in a cluttered scene [16,20,21,22]. The estimation of local geometric surface features is often a prerequisite in the segmentation pipeline and is useful in many other applications [23,24]. In 3D point clouds, the three most widely used methodologies to extract geometric features are Random Sample Consensus (RANSAC), 3D-Hough transform (3D-HT) [18,23,25], and region-growing [23].

Random Sample Consensus is used to classify building planar features from a 3D point cloud [20,26] due to its computational efficiency in outlier settings. Several variants of RANSAC exist to optimize execution speed and accuracy in higher outliers. Reference [20] enhanced RANSAC with an octree structure to detect multi-planar building facades, and in [27] the point cloud is subdivided into cells to detect minimal planar patches. However, RANSAC is highly sensitive to parameter tuning depending on the noise levels [14]. Hough transform (HT) is more prominent in detecting parameterized features in linear and circular shapes in 2-dimensions (2D) compared to 3D datasets [28]. To facilitate the speed and accuracy of HT in 3D settings, in [29] a randomized version of HT was developed where planes and curves are detected by mapping points into one point in Hough space and randomly voting points based on a distance from the candidate planes. In [28], different variations of 3D-HT were evaluated to detect planes from a simulated laser scan model, while in [30] HT was used to extract circular and rectangular columns from the projected points in the x-y plane; however, the implementation of 3D-HT is still disfavored due to its overall high computation cost [15,28]. As demonstrated above, both RANSAC and HT are useful in detecting geometric shapes in noisy conditions; however, both techniques have been shown to underperform in large datasets, and often tend to create spurious planes [15].

Region-growing is also widely implemented to detect geometry features in the point cloud scenes of buildings due to its high resilience in contaminated data [13,16,18,21,23,24,31]. The main limitation of region-growing is the selection of a seeding point that can affect the quality of segmentation [32]. Also, during clustering, the algorithm can result in the over- or under-segmentation of regions leading to the spontaneous creation of region borders [32]. Due to these problems, hybrid [32] and clustering integrations [18] are suggested to overcome these limitations. In [33], the region-growing was modified with a smoothness-constraint approach where close points with similar surface curvature are merged. This approach is deployed in the point cloud library (PCL) [34] and applied in [13,16] to detect planar patches. More recently, robust methods of Principal Component Analysis (PCA) have also been applied to segment planes in the presence of a high proportion of outliers. In [24,32], a statistical-based PCA that uses a fast-minimum covariance determinant to extract multiple planar objects from 3D point sets was applied. Nevertheless, the mentioned approaches are yet to solve the problem of missing data due to extensive occlusions and separate nearly identical structural and non-structural elements in a point cloud scene. 

### 2.3. Clustering and Segmentation Approaches

To overcome the limitations of missing data and noise, we adopt the spatial sub-division strategy where the point cloud is empirically decomposed to detect approximate coplanar patches at the voxel level and merge patches with similar saliency features in adjacent voxels. The use of octree-based segmentation has proven to be computationally efficient and insensitive to outliers [13,14,15]. 

The segmentation of structural elements from a raw point cloud is a challenging task due to clutter and the presence of occlusions that are diverse in nature. As a solution to this, recent clustering methods have integrated the local spatial features and the contextual knowledge of building components in their segmentation frameworks. In [16], the local concavity and convexity properties of building features were used to automate the as-built 3D remodel. The authors isolated scenes of interest by using material color properties in RGB scale. In [12,22,24,26,35], a histogram of height variation was used to classify horizontal components. These studies have exclusively experimented in construction-related environments where most of the structural components are less occluded compared to existing (occupied) buildings. In [12], this method was extended by creating z-bins based on discrete point densities. Although this approach is computationally efficient, it can be ineffective to apply in highly cluttered scenes with close variations of point densities, as well as in segmenting non-horizontal elements. In [36], the density-clustering approach was modified by obtaining a local density within a cutoff distance using a proposed exponential function. This model was applied to extract the wall points from the boundary points and segment the floors and ceilings using the established local density and z-values. As for other density-clustering models, this approach has been shown to underperform when objects are close to each other [36]. 

### 2.4. Deep Learning Application in Semantic Segmentation

Following the developments of baseline deep learning methods such as PointNet [37], PointNet++ [38], and dynamic graph convolutional neural network (DGCNN) [39], several studies have applied and modified these techniques to segment objects from the point cloud scenes. In [40], PointNet was used to train datasets and automatically detect and classify the structural components of bridges. In [41], a Surface Normal Enhanced PointNet++ (SNEPointNet++) was detected to semantically segment various defects in bridges. Since PointNet does not integrate the local structures of the point cloud [42], datasets in [17] are trained on the DGCNN model to learn local features on point neighborhoods and later extract planar patches and classify them into structural components. The authors tackled the problem of missing data by updating the network of patches with an incremental increase in the size of voxel units. Moreover, elements with insufficient planes were modeled using the dimensions of the adjacent similar elements with sufficient planes. The downside to this approach is that it performs in settings containing a repetition of elements with similar dimensions and may fail to segment elements with relatively larger missing data.

Though deep learning models have been tremendously resourceful in reducing the unnecessary time for processing point clouds [42] and offering great benefits in transfer learning [10], they still have some drawbacks. Generally, they demand large numbers of training datasets, which can be computationally expensive [42], and they struggle to perform both semantic and instance segmentation in some complicated scenes [42]. In the study conducted in [10], PointNet has demonstrated low performance in classifying standard building components such as ceilings, floors, and walls when no color attributes are used despite the great performance it has shown in classifying other indoor objects such as chairs, tables, and bookshelves.

To address the abovementioned challenges, we developed a training-free approach that can perform in complex scenes without the use of RGB information. The approach is constructed to be able to distinguish structural components from other objects separated by small offsets. Novelty methods are introduced on how to arrange pairs of detected planes and classify them accordingly with respect to the global representation of structural elements. Objects with insufficient planes are independently segmented without referring to the dimensions of other objects in the scene.

## 3. Methodology

In this study, we develop a robust method to segment large point clouds into accurate representations of structural elements in settings with high clutter and occlusions using the spatial dependency of structural forms. The spatial and programmatic qualities of structural forms have huge potential in analyzing load-bearing structures [43], and in the current research we apply the concepts of the spatial and functional connectivity of structural elements to segment the point cloud.

### 3.1. Overall Methodology

The study involves an innovative approach to extract planar surface patches from the raw and noisy point cloud and iteratively merge coplanar surface patches that appear to be scattered and disconnected from principal patches due to occlusions in the scene.

For efficiency, we deploy an octree-based segmentation approach where the input point cloud of points *P* = {*P_i_* ∈ ℜ^3^} is spatially subdivided into smaller cells. We detect planar patches in octree cells, followed up by the recursive merging of planar patches between the adjacent cells. The discretization of point clouds into smaller 3D data structures is computationally useful due to the spatial redistribution of the points in denser areas [13] and reducing the expensive neighborhood search using cells instead of points [15].

The segmentation of planar surfaces is performed through two steps: the merging of adjacent planar patches and the merging of scattered coplanar patches. The merging of adjacent patches involves the joining and clustering of minimal planar patches contained in adjacent cells. Then, among the clusters of merged patches, we select principal patches using a pair-wise parallel patch approach as reference patches for searching scattered and disconnected coplanar patches. The searching process consists of spanning the relative planes of principal patches and empirically assigning the disconnected patches as coplanar patches.

**Figure 2:**
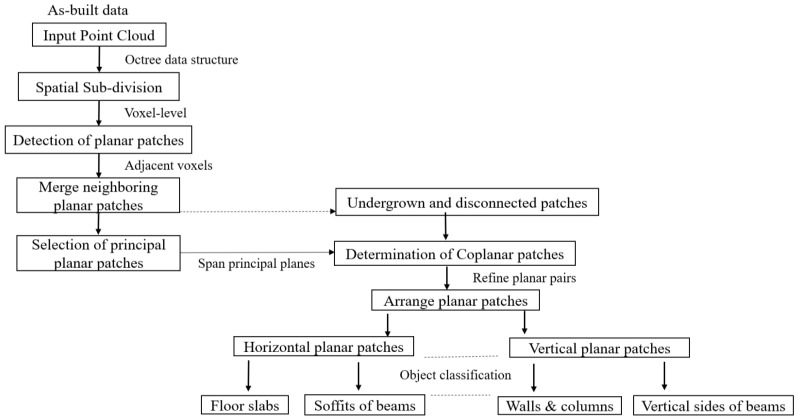
Overview of the developed approach.

After all the related coplanar patches are successively assigned and clustered, we arrange the pairs of planes in their respective orientations relative to the local coordinate system. The classification of planes is performed using the defined principles of structural forms and the spatial correlation of structural elements. The classification is based on the identification and segmentation of planar patches into forms of generic load-bearing elements defined in this study, which are slabs, beams, solid walls, and columns. Figure 2 illustrates the workflow of the proposed method.

### 3.2. Spatial Subdivision of the Point Cloud

The input point cloud is partitioned into voxels using an octree structure. An octree is selected as our spatial-subdivision data structure due to its inherited control over node partitioning compared to the KD-tree search [32]. As narrated in [44,45,46,47], an octree tree structure involves the recursive partitioning of the 3D data space into smaller volumetric units, where the structure divides the internal nodes into eight children. The partition approach for this study is inspired by the method implemented in [48] where the subdivision is coarsely performed until a certain number of samples, ϵ, in a voxel is reached. We give importance to an optimum number of points in voxels since sample size has a great bearing on the correlation structure of points [49], whereby having a very small sample size would lead to an erroneous data estimation and over-fitting, i.e., model skewing towards one datum [50], while a too large sample size would otherwise lead to diminishing returns [51].

**Figure 3:**
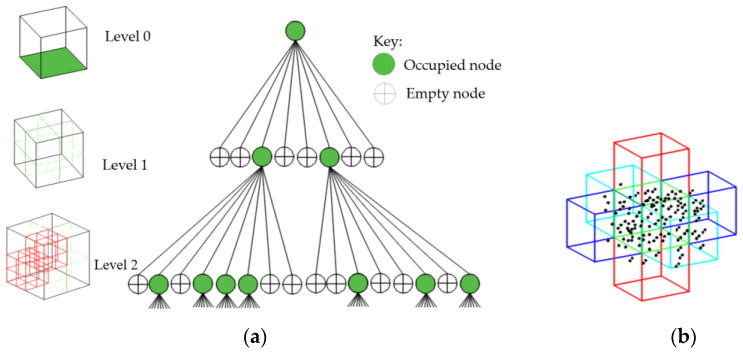
Point cloud subdivision: (**a**) octree structural framework; (**b**) abutting voxels for joining proximate planar patches.

Partition is initialized from the entire point cloud as the root node and coarsely subdivided into eight child octants as illustrated in Figure 3a, which proceeds to decompose until ϵ-points are reached. For this case, we recommend ϵ = 30, which is proven to be suitable for large point clouds as demonstrated in [48].

### 3.3. Planar Patch Detection

This subsection consists of the extraction of planar patches from the octree cells generated in Section 3.2. We use an octree-based region growing method in which we estimate the saliency features of ϵ neighboring points contained in voxels to detect the underlying planar patches and sequentially merge patches with similar features from the adjoining voxels. We adopt this method from [15], but we extend the method to find the remote coplanar patches as described in Section 3.5.

Principal Component Analysis is used to estimate the saliency features of points in voxels based on the covariance structure of local neighborhoods [52]. The principal direction of point distribution is determined from the neighborhood of ϵ points contained in voxels to extract minimal planar patches. Plane fitting is performed in voxels by estimating the plane parameters using normal vectors, $\vec{n}$, and a residual value, *r*. Normal vector, $\vec{n}$ relative to the smallest eigenvalue, λ_0_ of the covariance matrix **C**, are ordered by decreasing eigenvalues λ_2_ ≥ λ_1_ ≥ λ_0_ ≥ 0, with the corresponding eigenvectors v_2_, v_1_, and v_0_. The set of neighbors around the point *P_i_* is obtained, and the covariance matrix (**C**) around the neighborhood mean P is computed in Equation (1) [10].

(1) $$C=\frac{1}{n-1}\sum_{i=1}^{n}\left(\mathrm{Pi}\;-\bar{P}\right){\left(\mathrm{Pi}\;-\bar{P}\right)}^{T}$$

**Figure 4:**
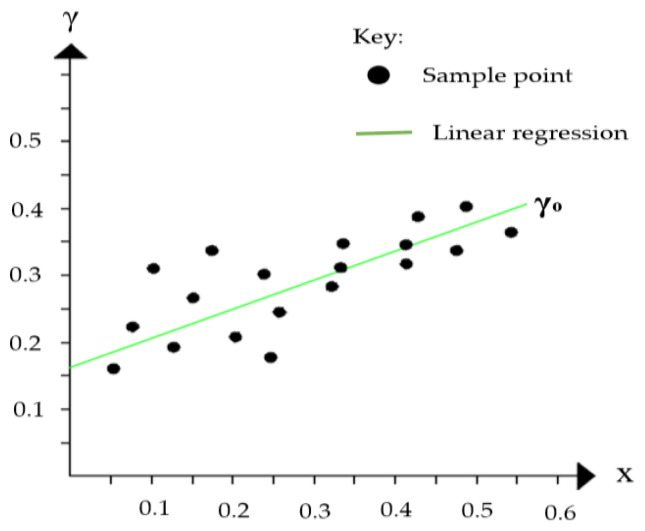
Point distribution around the fitted plane on the x-y plane, and the estimated root mean square error.

As described in [15], the residual value, *r* describes the distances of each point, *Pi*, from the best-fit plane, ℿ, as shown in Figure 4 and calculated in Equation (2). The value of *r* relates to the noise errors and deviations in the neighborhood of points as explained in [33].

(2) $$r=\sqrt{\frac{1}{N}\sum_{i=1}^{N}{\left(\gamma -\gamma_{0}\right)}^{2}}$$

As applied in [15,48], the points in each voxel are arranged in the order of decreasing planarity relative to the eigenvalues. The most planar point is then selected as a seeding point and grows coarsely to the nearby points that have similar surface normals. The merging process stops when no more points can be added to the patch. Points in the patches are assigned and the best-fit plane (ℿ) is expanded on the neighborhood. This method returns a set of *k* minimal planar patches, *Ś_i_* = {*Ś*_1_, *Ś*_2_, …, *Ś_k_*}.

### 3.4. Merging Planar Patches

**Figure 5:**
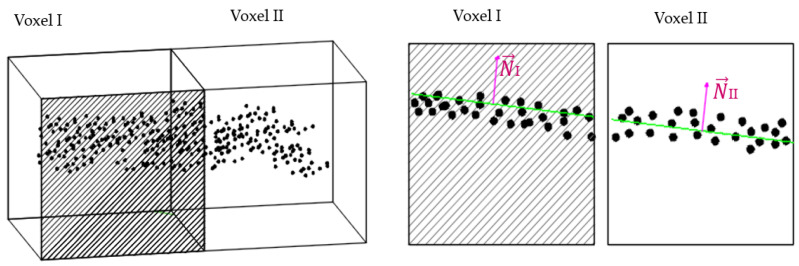
Adjoining voxels containing planar patches with similar normal orientations as candidates for the merging.

The extracted planar patches (*Ś_k_*) are merged together in their immediate proximate cells if they share similar saliency features [15], as illustrated in Figure 5. In this case, neighboring patches with similar normal orientations ($\vec{n}$) are considered candidates for merging. Once patches are merged, we re-fit the new plane by the least-square method [53]. Afterward, a refinement process is performed to cluster the unassigned points and test for plane validity using a pre-conditioned parameter based on the measured noise level. Details of the refinement process can be found in the literature [15].

The newly merged planar patches *S_i_* = {*S*_1_, *S*_2_, …, *S_n_*} are classified into two clusters based on the sizes of the patches. Herein, we use the surface area of a patch, *A_s_*, to define the size instead of the number of points due to expected variability in point densities. Patches with surface areas (*A_s_*) equal to or more than 0.15 m^2^ are labeled as complete (in our experiment) and clustered together in a set, *R_i_* = {*R*_1_, *R*_2_, …, *R_n_*}. The rest of the patches are labeled incomplete (undergrown) and clustered in a set *Ŕ_i_* = {*Ŕ*_1_, *Ŕ*_2_, …, *Ŕ_m_*}. Points left unassigned after the refinement process are also appended in a cluster *Ŕ*. We set the threshold value *A_s_* ≥ 0.15 m^2^ to represent the expected minimum detected surface of the structural element.

However, this approach only merges planar patches based on their proximity defined by their abutting voxels as shown in Figure 3b, and in the condition of high occlusions, which is common in scenes of occupied buildings, other coplanar patches or points can be distant [17]. This phenomenon is often described as missing points, which may lead to some coplanar patches being scattered and disconnected. The next sub-section counters this challenge by searching for candidate coplanar patches/points.

### 3.5. Search for Remote Coplanar Patches and Points

**Figure 6:**
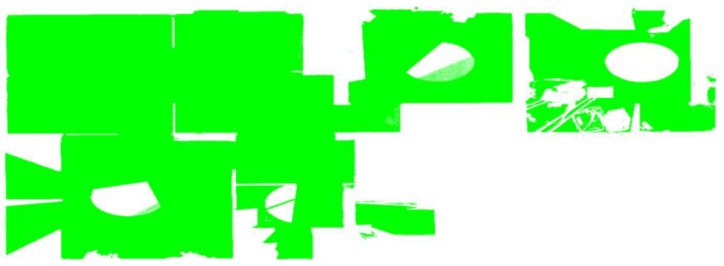
Gaps on planar surfaces due to missing data caused by extensive occlusions.

Some studies have tried to solve the problem of missing data during the segmentation of point clouds. In [17], the size of voxels was enlarged to find overlaps between planar patches to approximate disconnected coplanar planar patches. In [32], a predetermined value was used to estimate the least difference in Mean Square Error (DMSE) between the neighboring regions with similar features to merge surface patches. Despite their successes, both methods have relied on patch proximity. These approaches are difficult to apply in scenes with high occlusions, which can result in sizeable missing data disconnecting coplanar patches as illustrated in Figure 6.

**Figure 7:**
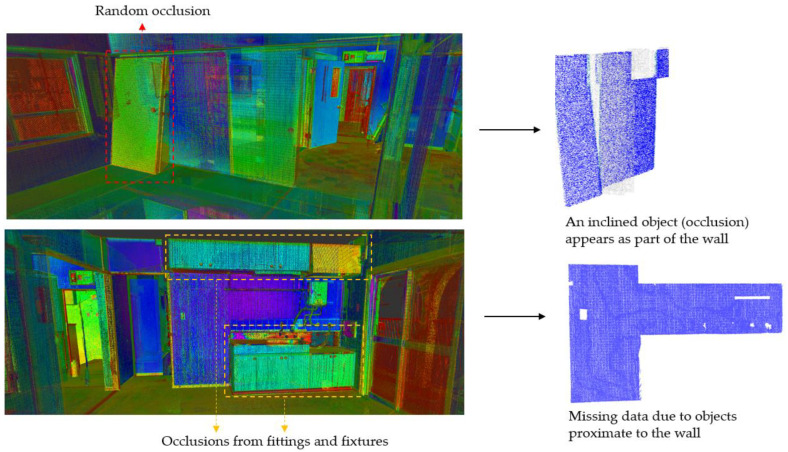
Presence of occlusions along the walls.

Point cloud acquisition is prone to noise, which can be *scene-related* or *device-related* noise, where the former consists of unwanted objects found in the scene and the latter pertains to sensor limitations and errors during the acquisition and post-processing of the point cloud [14]. Noises due to devices are usually estimated with reference to instrument specifications, while scene-related noises are difficult to measure due to the heterogeneousness of clutter. Noises due to non-permanent and permanent objects present in indoor scenes can obscure the segmentation process of structural elements especially when they are in the vicinity as demonstrated in Figure 7.

To address the issue of large missing data and the surface affinity between the structural and non-structural objects, we propose a novel approach consisting of linearly interpolating the undergrown patches and unassigned points (*refer cluster Ŕ*) using *patch-pair correspondence*, i.e., principal patches. The principal patches are empirically drawn from the set *R* based on the *patch-pairing* technique as described in Section 3.5.1. The use of *patch-pair correspondence* helps to introduce the segmentation attribute relating to the surface topology of structural components that appear in pairs of parallel planar faces.

Afterward, we span the planes fitted in the principal patches and deploy them as *proxies* to identify the candidate coplanar patches and points using the statistical inferences from the known samples. We further check for the presence of outliers and validate for coplanarity for further processing.

#### 3.5.1. Determination of Principal Patches

In this subsection, we deal with patches from set *R* (*R_i_* = *R*_1_, *R*_2_, …, *R_n_*) to determine the principal patches. At this point, there is a considerable number of undesirable planar patches from the clutter, and we initialize the filtration process by uncovering sets of *paired-parallel* patches. The essence of pairing the patches is to infer the surface topology of generic structural elements bounded by pairs of opposing surfaces in a given distance relative to the standard thickness/depth of the elements.

**Figure 8:**
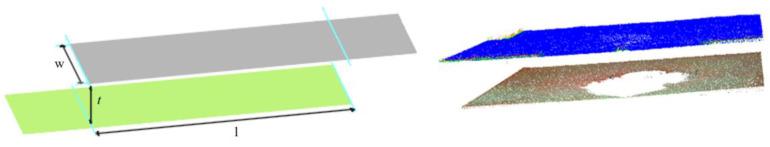
Alignment and overlap of a pair of parallel planar patches.

From each patch (*R_i_*) we extract the following feature parameters: Normal vectors ($\vec{n}$*_i_*), Area covered by the patch (*A_s_*), and Centroid (*C_i_*). These parameters are applied in combination with conditions related to the offset distance between two parallel planes and differences in patch sizes and overlaps are used to determine the patch pairs. The in-depth procedure of this approach is described below:

*(a)*  The orthogonal distance and angular value between the approximate parallel patches
  A tolerance (*t*) is used to define the maximal orthogonal distance between the two parallel patches, which is equivalent to the standard thickness of common structural elements. The value of *t*, where *t* ≤ 50 cm between the two neighboring parallel planar patches, is used to assign the candidate pairs of parallel patches. For two patches to be parallel, we consider the maximum angular value of not more than 5° between their normals ($\vec{n}$*_i_* & $\vec{n}$*_i_*_+1_).
*(b)*  Patch sizes
  This part deals with the comparison of patch coverage by their surface areas. The two parallel patches (*R_i_* & *R_i_*_+1_) with surface areas (*l × w*, measured on their extreme edges), *A_s(i)_* and *A_s(i_*_+1)_, respectively, are compared against one another to enhance the picking of true patches representing structural elements as opposed to occluding objects in the vicinity.
  Given two adjacent and parallel patches, we set a condition regarding a difference in their surface areas (*d_A_*) using Equation (3), such that the ratio *d_A_*/*A*_*s*(*i*)_ or *d_A_*/*A*_*s*(*i*__+1)_ (*whichever is larger between A*_*s*(*i*)_ and *A*_*s*(*i*__+1)_) is not more than 20%. We opted to use the surface areas instead of the number of points in a patch, which is influenced by point density that varies depending on the distance between the sensor and an object [33].
  d_A_ = |A_s(i)_ − A_s(i+1)_| (3)
*(c)*  Patch overlap
  In consideration of Section 3.5.1a,b, which only deals with the adjacency and matching sizes between two parallel planar patches, however, the two patches may still represent surfaces from two different objects. To solve this, we introduce a patch-alignment criterion whereby the two parallel patches have to coherently overlap by more than 50% of their surface areas (*A_s_*) as displayed in Figure 8.

The overall procedures in Section 3.5.1a–c return a set of *n* pairs of parallel patches referred to in this study as the *principal patches*, *Ṗ*, for *Ṗ_n_* = {*ṗ*_1_, *ṗ*_2_, …, *ṗ_n_*} and their associated fitted-planes (ℿ).

#### 3.5.2. Spanning of Principal Planes

Given a set of principal patches (*Ṗ_n_*), we span their corresponding planes (ℿ) and locate the disconnected and remote coplanar patches, spanning a plane (ℿ) where the *Span*(ℿ) ∈ ℜ^3^ is initialized by finding a linear combination, *W*, as shown in Equation (4) for a non-empty set of vectors $\vec{u}$*_i_* = {$\vec{u}$_1_, $\vec{u}$_2_, …, $\vec{u}$*_n_*}.

(4) $$W\;=C_{1}{\vec{u}}_{1}\;+\;C_{2}{\vec{u}}_{2}\;+\;\ldots \;+\;C_{n}{\vec{u}}_{n}$$

For some scalars *C*_1_, *C*_2_, …, *C_n_*, given that $\vec{u}$_1_, $\vec{u}$_2_, …, $\vec{u}$*_n_* is a set of non-parallel and linearly independent vectors.

Planes (ℿ) are spanned in all the directions of vectors $\vec{u}$*_i_* by adjusting the scalars in ±*C_i_*. The *Span*(ℿ) terminates at each local maxima and minima of the input point cloud, encompassing all the xyz components. We also use them in setting the bounding box. Placing the spanning constraints helps to avoid the unnecessary computations of infinity planes (ℿ_α_), which reduces the search for approximate coplanar patches and/or points.

#### 3.5.3. Points Assignment to Principal Planes

This section deals with assigning points and undergrown patches from the *cluster Ŕ* onto the spanned principal planes (*Span*(ℿ)) to determine the remaining coplanar patches/points. To initialize the process, we calculate the orthogonal distances of each point (*ρ_i_*) in the cluster *Ŕ* (*ρ_i_* = {*ρ*_1_, *ρ*_2_, …, *ρ_n_*}) to the closest *Span*(ℿ*_i_*) in ℜ^3^ in order to preliminarily find the candidate coplanar points. Points falling within the allowable distance threshold are assigned to the corresponding plane, *Span*(ℿ*_i_*). Afterward, the assigned points are subjected to testing for outliers and their coplanarity. The following discussion covers a description of the process:

*(a)*  *Points-to-Plane-Approximation*

Points (*ρ*) in the *cluster Ŕ* are drawn and measured in their offset distances to the spanned planes. We start the process of approximating point-to-plane by defining a distance, *f*: *ρ* → *Span*(ℿ*_i_*), such that *f* is the orthogonal distance of point *ρ_i_* to a spanned-plane (*Span*(ℿ*_i_*)), within a pre-signed allowable distance (δ*_dist_*. The distance (*f*) of an arbitrary point, *ρ_i_* = (*x*_1_, *y*_1_, *z*_1_); *ρ_i_* ∈ ℜ^3^, to a plane represented in general form: Ax + By + Cz + D = 0 where (A, B, C) denotes the unit normal vector, $\vec{N}$, is computed as Equation (5);

(5) $$f=\frac{\left|\mathrm{Ax}1+\mathrm{By}1+\;\mathrm{Cz}1\right|}{\sqrt{A^{2}+B^{2}+C^{2}}}$$

The threshold value, δ*_dist_*, is used to set a tolerance distance of a candidate sample to the plane, which is partially related to the concept of the *maximum distance* of sample-to-plane (*MDP*) applied in [14,54]. To determine the value of δ*_dist_*, we infer the sample distribution from the principal patches (*Ṗ_n_*) using the median estimator. The median estimator is deployed due to its resilience to outliers compared to the mean value [55].

Given a set of *Q* points (*Q* = *P*_1_, *P*_2_, …, *P_n_*) of the particular principal patch (*Ṗ_i_*) and the corresponding fitted plane (ℿ_i_), we compute *point-to-plane* distances, forming a set of measurements, Đ = {*đ*_1_, *đ*_2_, …, *đ_q_*} computed using Equation (5). To incorporate the noise errors, we adopt the use of *median absolute deviation* (*MAD*) to estimate the deviation of points around the plane as applied in [14]. From the set Đ, we first computed the MAD using Equation (6) and use it to calculate the value of δ*_dist_*. δ*_dist_* is designed to set the maximum limit over the median value using the standard deviation (*σ*), which is equivalent to MAD in our case as shown in Equation (7).

*MAD* = *Ω* × *median* (|*p*_*i*_ − *median*(*Đ*)|) (6)

where *P_i_* represents points in the set Q and Ω = 1.4826, which is the constant value in the normal distribution for the consistent coherence between the MAD and standard deviation values [48].

δ_*dist*._ = [*Median*(*Đ*) + *ƿ*
*MAD*(*Đ*)] (7)

For a probability distribution, we use the second quantile (2σ), *ƿ* = 2, as shown in resulting in a confidence interval of 95.45% to select true samples (inliers) around the median.

**Figure 9:**
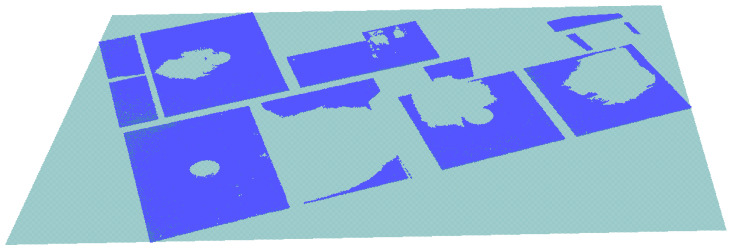
Disconnected coplanar regions as the result of occlusion present in the scene.

Points found within the δ*_dist_* are assigned to the corresponding plane (*Span*(ℿ*_i_*)) as *approximate coplanar points*. Moreover, points lying between the pair of principal planes and within the δ*_dist_*, are assigned to the nearest plane. The output of this procedure is the number of disparate and multi-density regions of points over the course of *Span*(ℿ) as shown in Figure 9. The next step consists of testing outliers in regions using the density-clustering approach.

*(b)*  *Outlier testing*

In the raw point cloud dataset, the measurement of noise errors is commonly used to detect outliers by setting the allowable offsets of samples-to-plane. However, it is difficult to measure the noise levels for raw 3D point clouds, as it is biased toward the sensors used and the noises may contaminate all three components (xyz) of the coordinate system [56]. Our approach is independent of the sensor’s noise levels; instead, we apply the knowledge acquired from the dataset. We use the statistical distribution and neighborhood structure of the samples to eliminate the outliers. Intuitively, the identified approximate coplanar points detected in Section 3.5.3a form *K* number of sparse regions across the *Span*(ℿ*_i_*). For this reason, we used the *Density-Based Spatial Clustering of Applications with Noise* (DBSCAN) approach to detect the outliers because it does not consider the number of clusters [57], and also it has good efficiency on large datasets [58].

In applying the DBSCAN, one of the fundamental challenges is finding the required input parameters [59]: the radius for *έ*-neighborhood (*eps*), and the minimum number of points within the *έ*-radius (*MinPts*). We used the approach in [60] to explicitly obtain the input parameters relative to the variation of density in clusters. Several values of *eps* are obtained for different densities in accordance with the *k-dist plot* and the *MinPts*, which are subsequently calculated for each *eps* as shown in Equation (8):

(8) $${MinPts}_{i}=\frac{1}{n}\sum_{i=1}^{n}Pi\;$$

where *P_i_* (*i* = 1, 2,…, *n*) is the number of points in the *eps* neighborhood for point *i*. Points left *un*-clustered are identified as outliers and eliminated. Clusters created in this step, *Ƈ_K_* = {ƈ_1_, ƈ_2_,…, ƈ*_k_*}, are assembled and tested if they align on the same plane as the principal patches.

*(c)*  *Coplanarity testing*

For each cluster detected, we apply PCA to determine the principal direction of the samples. Similar to the approach in Section 3.3, the covariance matrix, **C**, of neighboring samples, *N*, in the cluster (*Ƈ_i_*) decomposes and returns three eigenvectors $\vec{v}$_0_, $\vec{v}$_1_, $\vec{v}$_2_, and their corresponding eigenvalues *λ*_2_ ≥ *λ*_1_ ≥ *λ*_0_. The plane is expanded along the direction of the vectors representing the highest variation, i.e., $\vec{v}$_1_, $\vec{v}$_2_. Eigenvector, $\vec{v}$_0_ defines normal vectors $\vec{n}$^c^ of clusters.

Then, the orientations of the cluster’s normal, $\vec{n}$^c^, are matched with the principal plane’s normal, $\vec{n}$. If the angle between the two normal vectors ($\vec{n}$^c^ and $\vec{n}$) is within a maximal threshold of 2° (in our experiment), the cluster is then designated as a *coplanar patch*, or else the cluster is then deemed to represent another surface. The process continues across all spanned planes and their relevant patches.

### 3.6. Refining Pairs of Principal Patches

After the coplanar points are distinctly assigned to the corresponding principal planes (*Span*(ℿ)), the planes are subjected to a refinement process that involves matching pairs of planes prior to classifying them into the respective structural elements and each pair of the spanned principal planes, i.e., *Span*(ℿ*_i_*) & *Span*(ℿ*_i_*_+1_), is traversed to determine the number of points and their associated patches (clusters). Then, we compared the points coverage between the planes in each pair set using the the condition in Section 3.5.1b. The qualified pair sets are then subjected to a plane classification procedure as described in Section 3.7.

### 3.7. Plane Classification

Once the planes containing the patches (ℿ) are implicitly grouped in pairs, we now locally associate the planes with the structural elements in consideration: horizontal elements (floors, suspended slabs, and beams), vertical elements (walls and columns), and slanting members. First, we arrange the plane pairs according to their normal orientations ($\vec{n}$) relative to the local coordinate system such as:

- Horizontal Planes, ℿ*_h_* (including nearly horizontal planes): comprises pairs of planes with their normal vectors, $\vec{n}$*_h_*, oriented at an angle (Ɵ) < 45° to the *z-axis*;
- Vertical Planes, ℿ*_v_* (including nearly vertical planes): comprises pairs of planes with normal vectors, $\vec{n}$*_v_*, oriented at an angle (Ɵ) ≥ 45° to the *z-axis*.

The set of two planes in a pair is denoted as *i* and *j*, i.e., ℿ_*h*(*i*,*j*)_ for horizontal pairs and ℿ_*v*(*i*,*j*)_ for vertical pairs. After all pairs of planes are arranged in their respective orientations, we start to classify the planes by matching their local feature properties with the physical (*global*) attributes of structural elements. The following features of local planes are used in the classification process: normal orientations ($\vec{n}$), centroids, Č, (*X_c_*, *Y_c_*, *Z_c_*), and the surface area of planes (*A_p_* = *l* × *w*). Attributes of the structural elements are defined in accordance with the general standards and specifications of reinforced structural forms.

#### 3.7.1. Floor Slabs

As defined in [61], reinforced concrete (RC) slabs are structural member panels, horizontal *or nearly so*, bounded between and supported by beams, columns, walls, or the ground. This section deals with the detection of interior floor slabs consisting of the ground floor and the subsequent upper (*suspended*) floor slabs using the available horizontal pairs of planes ℿ_*h*(*i*,*j*)_. For the purpose of this study, we express the slabs according to their floor levels starting from the *first floor* (ground level) and the pairs of planes are denoted in the following manner:

- ℿ_*h*(0,*j*)_, for planes representing *floors* (upper skin of the slab), where *j* = {1, 2,…, *n*} corresponding to the floor levels;
- ℿ_*h*(*i*,0)_, for planes representing *ceilings* (bottom skin of the slab), where *i* = {2, 1,…, *n*} at a corresponding floor level.

**Figure 10:**
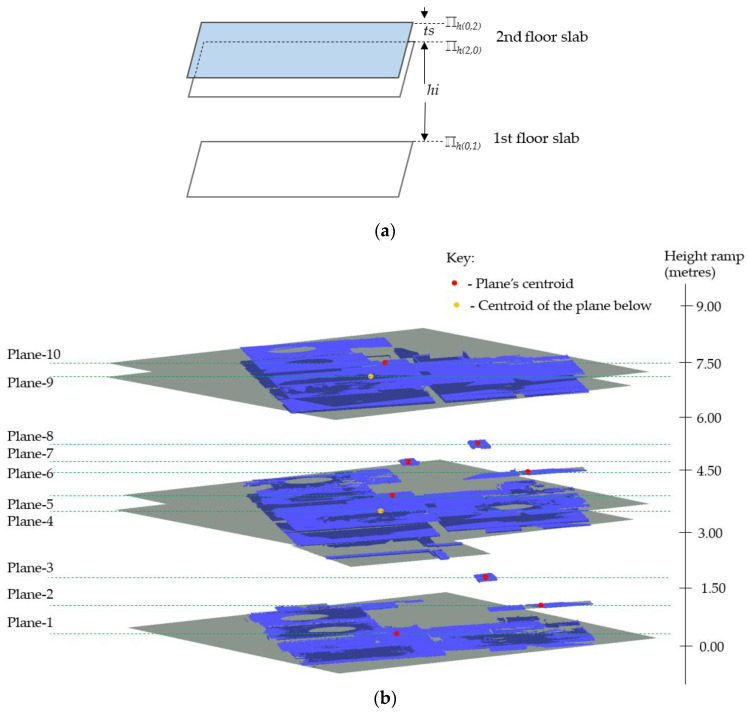
Vertical alignment of horizontal planes defining the respective floors and ceilings: (**a**) Planes representing the first- and second-floor slabs; (**b**) Horizontal patches including the detected occluding objects positioned along the z-axis.

To detect the floor slabs, we start by ranking the horizontal planes according to their spatial positions using the z-components to signify their global height placements. Since we are also dealing with the non-horizontal slabs, we use the z-components of the plane’s centroids (*Z_c_*) to represent the local-height positions of the planes. This process creates a z-order that comprises planes describing their elevation level with the lowest plane assigned to the *first floor*, (ℿ*_h_*_(0,1)_) as illustrated in Figure 10a. The subsequent floors are determined in ascending order as shown in Figure 10b.

The next step is to detect the *second-floor slab*, which involves identifying the associated planes representing the *ceiling* (ℿ*_h_*_(2,0)_), and the corresponding *floor* (ℿ*_h_*_(0,2)_). We prescribe criteria to determine the candidate planes for the ceiling and floor. We first set the orthogonal distance, *h*, between the *first-floor plane* (ℿ*_h_*_(0,1)_), and the succeeding parallel (*or nearly parallel*) plane in the z-histogram. The distance, *h*, stipulates the allowable minimum *story height*, where *h* ≥ 2.1 m for a standard residential building, and the succeeding parallel plane is labeled as the candidate ceiling plane (ℿ*_h_*_(2,0)_).

If the candidate plane (ℿ*_h_*_(2,0)_) conforms to the *h*-threshold, we further vet the plane in combination with its succeeding plane defined as the candidate plane for the *floor* (*Π_h_*_(0,2)_). The two planes (ℿ*_h_*_(2,0)_ & ℿ*_h_*_(0,2)_) are set to be within the predetermined distance (*t_s_*) stipulating the slab thickness (particularly for the *two-way slabs*), where *t_s_* < 50 cm.

In conjunction with the *t_s_*-threshold, the difference in surface areas (*A_p_*) between the two planes, ℿ*_h_*_(2,0)_ & ℿ*_h_*_(0,2)_ is set to be less than 15% (*in our experiment*). The area difference accounts for dissimilarity in surface exposure between the ceilings and floors, whereby floor beams conceal the ceiling surfaces as opposed to walls on the floors. Once all the conditions are met, the candidate planes ℿ*_h_*_(2,0)_ and ℿ*_h_*_(0,2)_ are eventually designated for the *ceiling* and *floor*, respectively, for the *second-floor slab* and the offset distance *h_i_* is signed as the first story.

The operation continues sequentially to detect the remaining floor slabs and their corresponding ceilings and floors. This approach helps to automatically obtain the respective story heights, *h_i_*, that we can use to segment the vertical components enclosed between the floors and ceilings, where *i* = {1, 2, …, *n*} for each *i* indicates a particular story level.

#### 3.7.2. Floor Beams

**Figure 11:**
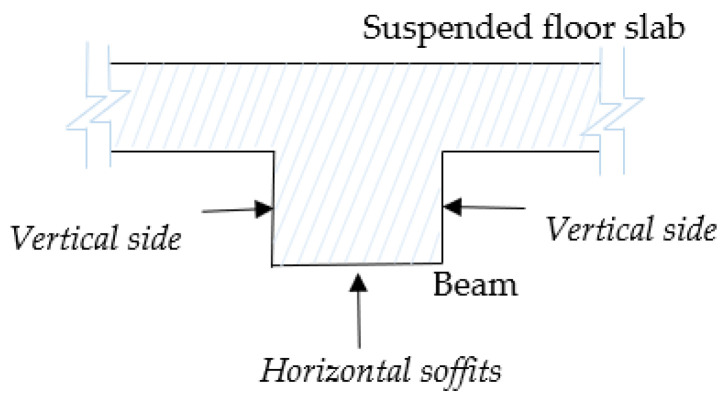
Cross-section of the concrete beam under the floor slab.

Concrete beams are composite structural members that transfer transverse loads from slabs to columns and/or load-bearing walls [62]. This study deals with the rectangular floor RC beams composed of three exposed planar faces, which are: the soffit of beams, which is the horizontal underside/ceiling of the beam, and the two parallel vertical sides as shown in Figure 11.

The planes for ceilings (ℿ_*h*(*i*,0)_) are used in this case to locate the surfaces of floor beams using the *ray tracing* approach. First, we determine the positions of the beam’s soffits by casting virtual rays on the ceiling’s plane. Rays managing to pass through the gaps on the ceiling plane are traced and locate the hitting points below the ceiling, which are further analyzed to represent the beam’s soffits. Thereafter, the patches representing the vertical sides of beams are determined.

**Figure 12:**
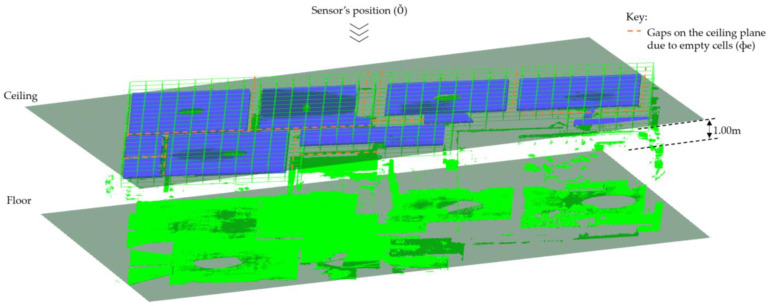
Alignment and position of the overhead ceiling and points below the ceiling plane for detecting candidate points for the corresponding beam below.

Initially, we search for points contained in cluster *Ŕ* and the remaining planar patches in cluster *R* (upon omission of the principal patches) that are found below the plane for the second-floor ceiling (ℿ*_h_*_(2,0)_). To extend the search process, we select only the points located 1.0 m below the ceiling’s plane as candidate points for the beam’s soffit on the second floor as presented in Figure 12. These points and the ceiling’s plane (ℿ*_h_*_(2,0)_) are used as the input dataset in the ray tracing operation.

The input dataset is subdivided in an octree framework into single-layered voxels [13] using a user-defined resolution (10 cm, in our experiment). This leads to the entire ceiling’s plane appearing in a rasterized structure similar to the approach in [63], containing *m* occupancy bitmaps, *ф*_i_ = {*ф*_1_, *ф*_2_, …, *ф_m_*}. The bitmaps on the plane are labeled as either *occupied*, *ф_¢_*, if it has at least one point, or *empty*, *ф_e_* if it has no point inside. Then, a series of *m* rays, *Ř_i_*, for *i* = {1, 2, …, *m*}, originating from the sensor’s position Ǒ (*above the ceiling’s plane*) are cast onto the input dataset and we compute the intersections made. If the ray does not hit any bitmap on the plane, we proceed to observe if any hit is made on the voxels below the *ceiling* plane in the direction of the ray. We identify the number of *k* voxels, *V_i_* = {*v*_1_, *v*_2_, …, *v_k_*} hit by rays below the ceiling and compute the distances of the intersection point and the source (Ǒ) using the hit function used in [64].

We extract *N* points from each voxel (*V_i_*) and fit planes using the least squares method [46] on the *έ*-neighborhood of *N* points, within a specified radius, which is equivalent to the estimated width of the beam (10 cm × *number of empty (ф_¢_) abutting bitmaps along the breadth*, in our experiment). As the result, a set of *n* horizontal patches are detected, *Ꝓ_h(i)_* = {*Ꝓ_h_*_(1)_, *Ꝓ_h_*_(2)_, …, *Ꝓ_h(n)_*}, representing the beam’s soffits.

Following that, using the planes for beam soffits (*Ꝓ_h(n)_*) and the associated ceiling plane, ℿ_*h*(*i*,0)_, we find the local positions of the neighboring pairs of vertical planes, *Ꝓ_V(i)_*, which correspond to the vertical sides of beams. A set of vertical *paired planes* (ℿ*_v_*) is used to determine the planes *Ꝓ_V(i)_* if they qualify for the following conditions:

- The normals, $\vec{n}$*_v_*, of a particular pair of vertical planes (ℿ*_v_*), should be perpendicular (*or nearly so*) to the normal direction, $\vec{n}$*_h_*, of a soffit’s plane, *Ꝓ_h(i)_*;
- The orthogonal distance between the pair of vertical planes (i.e., ℿ*_V(i)_ ↔* ℿ_*V*(*i*+1)_) is approximately equal to the breadth of the associated soffit’s plane, *Ꝓ_h(i)_*;
- The height of vertical planes (ℿ*_v_*) should correspond to the distance between the ceiling’s plane (ℿ_*h*(*i*,0)_) and the associated plane for the beam’s soffit (*Ꝓ_h(i)_*).

The pair set of vertical planes conforming to the above conditions are eliminated from the list of ℿ*_v_* and designated to represent the vertical sides of the floor beams. Henceforth, for each of the revealed beams, we unveil a set of three planes (*Ꝓ_h(i)_*, *Ꝓ_v(i)_*, & Ꝓ_*v*(*i*+ 1)_) representing the typical surfaces of beams under the floor ceiling.

#### 3.7.3. Walls and Columns

A set of vertical *pair-planes* (ℿ*_v_*) is primarily used to identify the points representing the surfaces of the walls and columns. In this research, we emphasize the columns and *load-bearing walls* construing a structural path starting from the floor slabs or beams and ultimately anchored to the floor. For the purpose of this study, columns shall also include the attached and isolated piers. A tolerance, *t_w_*, (10 cm ≤ *t_w_* ≥ 50 cm) is defined for wall thickness, where the value of *t_w_* = 10 cm is applied to avoid selecting non-structural walls such as drywalls.

We start the detection process by clustering the remaining vertical planes (ℿ*_v_*) into *vertical sub-spaces* where each plane is grouped into its respective floor (story) level. For each plane, ℿ*_v(i)_*, we use the plane’s *local z-maxima* and *z-minima* to determine at which floor level they are located based on the identified planes for the floors (*Π*_*h*(0,*j*)_) and ceilings (*Π*_*h*(*i*,0)_). Then, we use the observed story heights (*h_i_*) for each sub-space to set the required heights for the candidate wall planes (ℿ*_w_*). In this instance, we adopt the story height (*h_i_*) for the plane’s maximal height, *h_max_*, which applies to walls with heights stretching from the ceiling to the floor. For other walls, we set the minimum allowable height, *h_min_*, which is equivalent to the height from the beam’s soffit to the floor. The height *h_min_* is the difference between the story height (*h_i_*) and the depth of the corresponding floor beam (*h_d_*), as formulated in Equation (9). Therefore, for each *sub-space*, we identify the set of pair planes whose vertical elevations lie within the height thresholds (*h_max_* & *h_min_*) and the space between each pair-set translates to the distance threshold, *t_w_*.

*h*_*min*_ = *h*_*i*_ − *h*_*d*_ (9)

**Figure 13:**
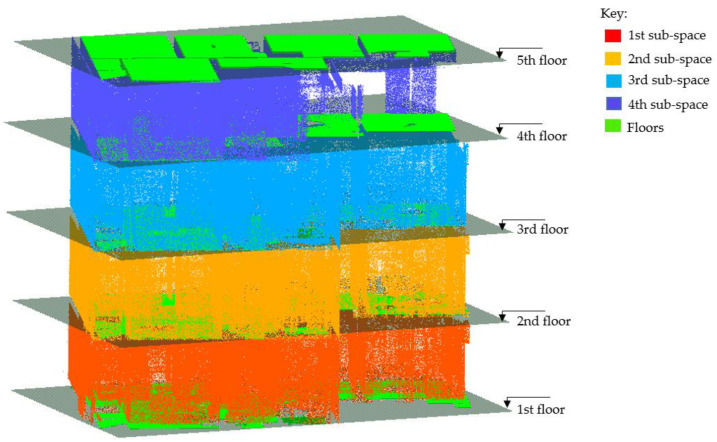
Vertical subspaces by floor levels.

This approach uses a *pair-wise* plane search, which is suitable for detecting internal walls that comprise a pair of planes that locally lie on the same *sub-space*. Henceforth, it can be ineffective in the classification of the external walls where one skin is in the interior and another is on the exterior of the building (façade), and the latter inherently do not fall within the floor partitions (*vertical sub-spaces*) as shown in Figure 13. To segment the planar surfaces representing the external walls, we first identify the planes for the façades (exterior skin) ℿ*_w(f)_*, and their corresponding interior planes, ℿ_*w*(*g*)_, separately, and pair them afterward using the thickness threshold, *t_w_*.

In principle, a common multi-story building has facades comprised of extended planar faces with disconnected surface patches due to large openings, recesses, protrusions [20], and occlusions from the external features. From the set *R*, we identify the remaining unpaired vertical patches and find the combination of coincidental planar patches, *ṝ_i_* = {*ṝ*_1_, *ṝ*_2_, …, *ṝ_n_*} and fitting planes accordingly. To identify the points representing the facades, we find the combination of *ṝ_n_* patches located beyond or along the edge points of the ceiling (ℿ_*h*(*i*,0)_) and floor planes (ℿ_*h*(0,*j*)_) in their x and y components. In doing so, we obtain the coplanar patches representing the exterior surfaces (facades) of the external walls across the perimeter of the building. Using the set *ṝ_i_*, we identify the planes that are perpendicular (or nearly so) to the planes for the facades (ℿ*_w(f)_*) within the thickness threshold (*t_w_*). The identified planes are then checked against the requirements for the wall height (*h_max_* − *h_min_*) and assigned as planes for the interior skin of the external wall (ℿ*_w(g)_*).

The walls are formed by two parallel planes (layers), while the columns are formed by two pairs of planes in the opposing direction. The length-to-thickness ratio (4:1) described in [65,66] is used to distinguish between the planes forming the walls and columns. The lengths of planes in the two pair sets not exceeding four times (i.e., 4×) the perpendicular distance between them (thickness) are classified as planes for columns. Those having lengths over 4× the thickness are classified as planes for walls.

## 4. Experimental Results

### 4.1. Overview

This section addresses the results obtained from the application of the proposed segmentation approach to a real dataset. We evaluate the efficiency of our approach using an unmodified large 3D point cloud with a high degree of noise and occlusions. The evaluation process is divided into three main parts. Firstly, we study the results obtained from the generation and merging of planar patches at the voxel level; secondly, we evaluate the performance of point assignment to the principal patches. Then, we analyze the quality of the classification of planar patches into the predefined structural elements, which are floor slabs, beams, walls, and columns.

The proposed method was implemented on Open3D 0.16.0, CloudCompare v2.12.3 (Kyiv, Ukraine) (64-bit), and Python 3.9.12. All the experiments were performed on a 3.70 GHz Intel(R) Core(TM) i9-10900K processor and 32 GB of RAM and an NVIDIA Geforce RTX 3070.

### 4.2. Dataset and Instrument

**Figure 14:**
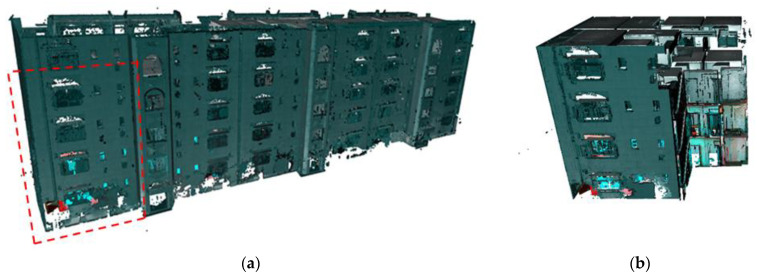
Input point cloud for the experiment: (**a**) Original point cloud; (**b**) Cut portion used in the experiment.

The dataset was obtained from multiple registered scans with known scan positions, encompassing the interior and exterior scenes. A Zoller + Fröhlich (Z + F) Imager^®^ 5016 terrestrial laser scanner (TLS) was used to scan a five-story apartment building consisting of a reinforced concrete structure as shown in Figure 14a. The technical details and specifications of the scanner are provided in [67]. The building comprised 30 fully furnished residential units with typical floor layouts, located in Onojo, Fukuoka.

The building was scanned from several scan positions to capture all the necessary surfaces resulting in an unmodified large point cloud. For experimental purposes, we randomly cut a portion of the original point cloud to retain a true representation of the building site with a manageable data size as shown in Figure 14a,b. The scanning results for these scenes are illustrated in Table 1.

### 4.3. Evaluation Metrics

For performance evaluation of our approach regarding the segmentation and classification of points into several classes of structural elements, we use the quantitative metrics *precision*, *recall*, *F1_-score_*, and the intersection-Over-Union (*IoU*), defined [11,68] as follows:

(10) $$\mathrm{Precision}=\frac{\mathrm{TP}}{\mathrm{TP}+{\mathrm{FP}}^{\prime }},\;\mathrm{recall}=\frac{\mathrm{TP}}{\mathrm{TP}+{\mathrm{FN}}^{\prime }},\;F1_{\text{-}\mathrm{score}}\;=\frac{2\mathrm{TP}}{2\mathrm{TP}+\mathrm{FP}+\mathrm{FN}},\;\mathrm{and}\;\mathrm{IoU}=\frac{\mathrm{TP}}{\mathrm{FP}+\mathrm{FN}+\mathrm{TP}}$$

where TP represents true positives, which refers to the number of class elements (slabs, beams, or walls) that were detected and found in the existing building; FP represents false positives, which refers to the number of detected class elements that were not found in the existing building; FN represents false negatives, which refers to the number of undetected class elements that were not detected by the proposed method. We also use true negative (TN) to evaluate our model’s ability to correctly predict the negative classes.

The number metric is used to measure and evaluate the floor slabs based on the amount of ceiling and floor surface revealed. For the floor beams and walls, we used the linear metric in running lengths to measure the quantities. This is due to the distinct nature of beams and wall layouts where they are represented in partial lengths between corners or columns and often occupy several arrangements and orientations dividing the floor or ceiling spaces. For the walls, the detected pairs of vertical planes are measured by their average extreme dimensions excluding the openings.

### 4.4. Preliminary Data Processing

Initially, the input point cloud is manually cropped to remove the unwanted objects appearing separated from the site of interest including nearby buildings, roads, and trees. Then, voxel-based down-sampling is applied to reduce the time for processing the data and sparse points in dense regions. The input point cloud *P*′ with 66,997,720 points is uniformly sub-sampled using a 0.05 mm voxel size, generating a point cloud *P* with 30,129,150 points, such that *P* ⊆ *P*′.

### 4.5. Generation and Merging of Planar Patches

#### 4.5.1. Spatial Sub-Division

**Figure 15:**
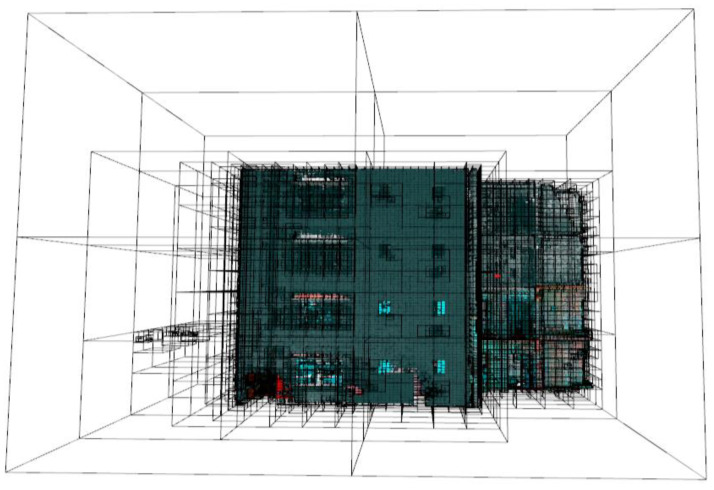
Spatial sub-division of the input point cloud.

The input point cloud *P* is spatially subdivided using a pointer-based region octree to produce a voxelized point cloud as presented in Figure 15. The decomposition of the point cloud is initiated at the internal node level 0 with 30,129,150 points into eight children and recursively decomposes guided by an early termination criterion, *ϵ*. The partition ends at the internal node depth 9. The partition produced 1,859,408 cells, each with an average population of 47.3671 points (±19).

#### 4.5.2. Planar Patch Generation

Features are extracted in the voxels using PCA and fit a plane in the principal direction of the points, whose normal is given by the smallest eigenvalue (*λ*_0_). The octree structure is traversed in all internal nodes until all planar patches are detected. This process generated about 1,487,525 minimal planar patches of distinct sizes. Non-empty voxels and voxels with fewer than 30 points were excluded from this process.

#### 4.5.3. Merging of Planar Patches

The planar patches from the adjoining voxels were merged provided that the difference in their normal vectors, $\vec{n}$*_i_* & $\vec{n}$_1+*I*_ lay within a maximal angular value of 5°. As a result, 1,175,146 detected planar patches were merged, which was equivalent to 79% of all the minimal planar patches. The remaining patches together with the merged patches with surface areas (*A_s_*) less than 0.15 m^2^ were clustered in *Ŕ_i_* = {*Ŕ*_1_, *Ŕ*_2_, …, *Ŕ_m_*}.

**Figure 16:**
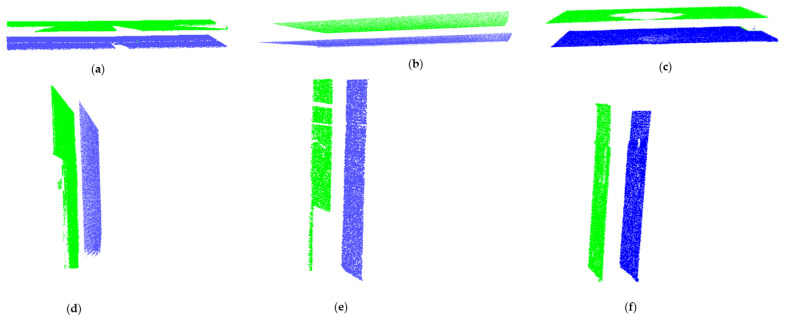
Pairs of generated principle planar patches: (**a**–**c**) horizontal principal patches; (**d**–**f**) vertical principal patches.

**Figure 17:**
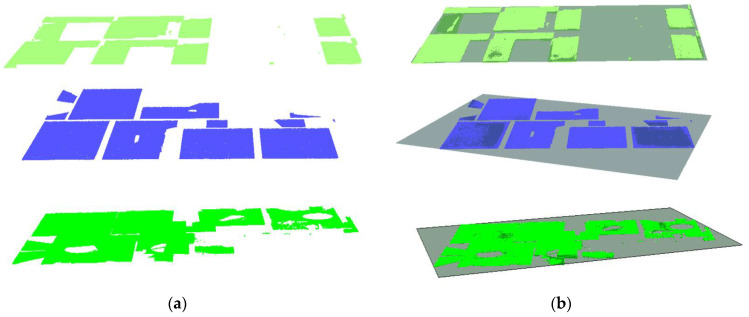
Results of spanning the horizontal principal planes and the subsequent assignment of disconnected coplanar patches: (**a**) disconnected principal patches; (**b**) planes fitted after spanning the principal patches and assigning the corresponding coplanar patches.

**Figure 18:**
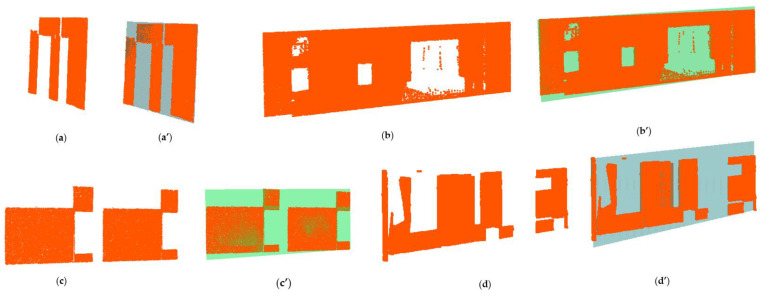
Results of spanning the vertical principal planes and the subsequent assignment of disconnected coplanar patches: (**a**–**d**) disconnected principal patches; (**a’**–**d’**) planes fitted after spanning the principal patches and assigning the corresponding coplanar patches.

Afterward, the pairs of principal patches were determined using the criteria shown in Figure 16. The principal patches were then spanned and the disconnected coplanar points and undergrown patches from the cluster *Ŕ* were assigned to the respective plane. Figure 17 and Figure 18 illustrate the spanned principal planes with their corresponding coplanar patches and points assigned to them.

**Figure 19:**
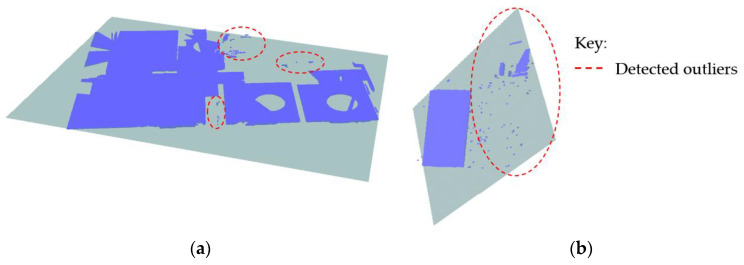
Point assignment to the spanned principal planes in searching for coplanar points and patches: (**a**) Outliers detected; (**b**) Surface patches and points from the clutter objects converge with spanned plane.

The point assignment to the spanned plane is followed up by the removal of outlier points and minimal clusters generated using the DBSCAN approach as shown in Figure 19a. The elimination of minimal clusters helps to avoid the inclusion of small clusters as coplanar regions as presented in Figure 19b since the assignment of points in the *Span*(ℿ) may overlook these regions.

### 4.6. Plane Classification

The principal planes (ℿ) are arranged and clustered into horizontal (ℿ*_h_*) and vertical (ℿ*_v_*) orientations pertaining to the conditions described in Section 3.7. Thereon, the planes are classified into the respective embodiments of structural elements in the following order: floor slabs, floor beams, walls, and columns.

#### 4.6.1. Floor Slabs

The horizontal planes (ℿ*_h_*) are deployed and selectively classified into floor slabs by sorting them into the corresponding surfaces representing the floors and ceilings. The parameter thresholds stipulated for story heights (*h_i_*) and slab thickness (*t_s_*) are used to subjectively provide the logical sequencing of surface skins characterizing the floor slabs (floor and ceiling).

**Figure 20:**
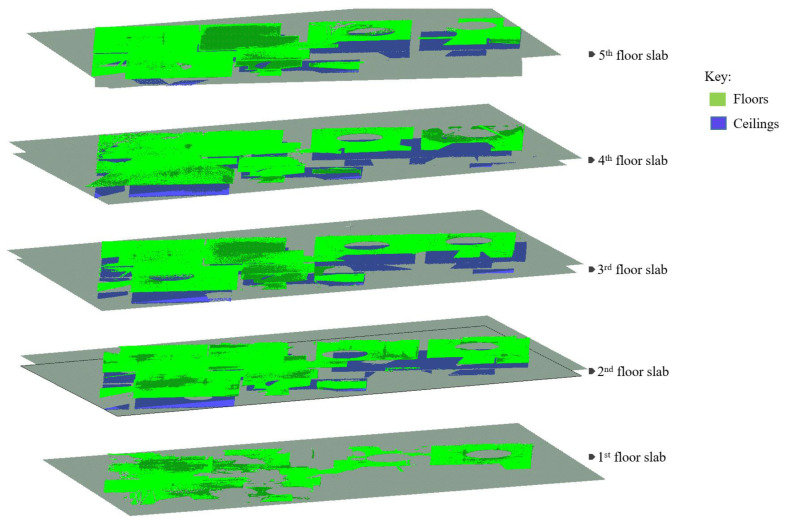
Floor slabs detected in the experiment are represented by the pairs of ceiling and floor planes.

As a result, we identified and clustered five horizontal planes corresponding to the floor surfaces and four horizontal planes for the ceiling surfaces, which together are equivalent to four-floor slabs as shown in Figure 20.

#### 4.6.2. Floor Beams

**Figure 21:**
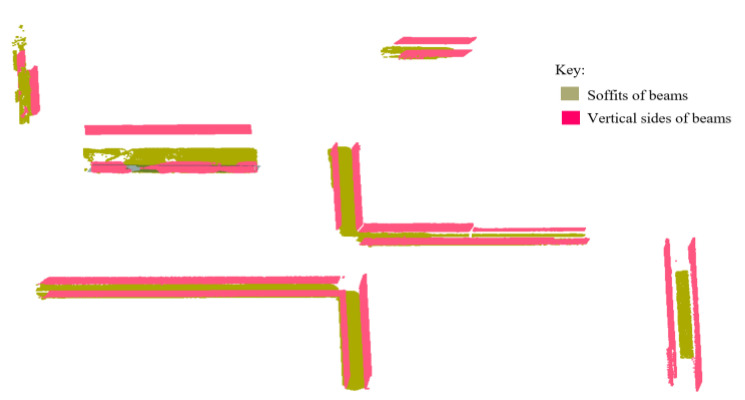
The detected soffits and vertical sides of the second-floor beams.

The methods proposed in this research are designed to classify surface patches and planes into floor beams through the detection of the three main surfaces of floor beams: *soffits* and the two opposing *vertical sides*. The beam’s soffits are detected from the *non-principal patches* in cluster *R* and the points remaining in cluster *Ŕ.* The ceiling plane is transformed into bitmaps and a series of rays are cast upon it to identify and track points within 1.00 m below it. The detected points are processed and emphatically designated as the beam’s soffits and are used to allocate the vertical pairs of planes representing the vertical sides of the floor beams as shown in Figure 21.

#### 4.6.3. Walls and Columns

**Figure 22:**
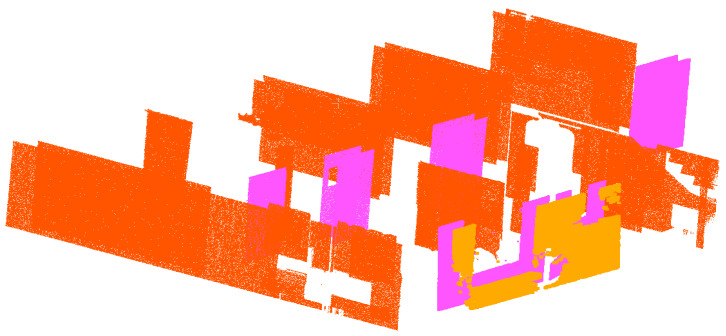
The detected walls from the 1st story.

The vertical planes (ℿ*_v_*) are used to detect the walls and columns found between the created vertical sub-spaces. The threshold *t_w_* is used to empirically separate the load-bearing walls and columns from other vertical objects including the light partitions and occlusions along the walls. As a result of the experiment, a total of 44 walls are determined and segmented. Figure 22 illustrates the walls detected from the first story.

## 5. Discussion

### 5.1. Evaluation of Points Classification

The quality of the classification process was evaluated by comparing the observed class elements from the experiment results against those of the ground truth inferred from the existing building. The quantitative evaluation of the classification of points into several elements (*floor slabs*, *beams*, and *walls*) was performed by using the four metrics: precision, recall, F1_-score_, and IoU. The IoU score is used primarily to bring the uniformity of relative errors due to the predominant occurrence of some class elements, in our case being the walls [11]. The results of the segmentation and classification process are presented in Table 2.

From Table 2, the floor slabs and beams are represented by their constituent surface planes, (i.e., the ceilings and floors for the floor slabs, and the soffits and vertical sides for the beams).

As Table 2 shows, all the ceilings and floors were detected with the proposed method, with a score of 1 in all the quantitative metrics. Due to that, all the floor slabs were detected with reference to the ground truth data. The first-floor slab appeared in one planar surface characterized as the floor surface, while the rest of the floor slabs were embodied in pairs of ceilings and floors. In the evaluation process, the ceilings and floors were considered in their full stretches and disregarded the size or number of gaps between the coplanar patches. The experiment was based on the interior slabs and the exterior recessed slabs only; the protruding balconies were ignored. The value of TN was raised due to the presence of a number of cabinet tops, which our models correctly identified as not floors.

The floor beams showed positive results, as shown in Table 2, amidst a high level of clutter on the ceiling surfaces. Beams as an overall element scored over 0.60 in terms of precision, recall, and F1_-score_, and as for the worst-case performance, our approach provided a score above 0.5 in IoU for detecting the floor beams. There was a slight difference accounted for the detected soffits and vertical sides of beams, which was produced by the presence of occlusions along the sides of the beams, especially in the openings. According to our approach, the vertical sides can only be detected in a pair set of vertical planes, so if one face is occluded it may affect the detection of both faces as a pair, which also hindered classifying the object as a beam and accounted for the FP value. Also, the detection of vertical sides is preceded by the detection of soffits; hence, the vertical sides are predestined to have lesser or equal scores compared to the soffits.

Some upper cabinets with breadths not exceeding 50 cm were falsely identified as beams and that affected the value of the FN. In addition, the overall detection of beams was affected by the surface exposed to the scanners. In particular, the isolated beams on the ceilings performed better in the detection process compared to the revealed beams with walls attached under them, and this is due to the difference in the extent of the exposed undersides (soffits) of the beams.

As Table 2 presents, the quality of our approach in classifying the walls also showed positive results. The performance of our approach in accurately not labeling the non-wall points as walls is presented with a precision score of 0.65, and the recall measure, which is related to the ability of our classifier [11] to find all the wall samples, recorded a score of 0.83. The worst-case performance of our segmentation and classification approach is marked at 0.58 IoU, which is still acceptable. Our approach showed an overall good performance in classifying the wall elements despite the substantial amounts of temporary and fixed objects on the faces of the walls.

**Figure 23:**
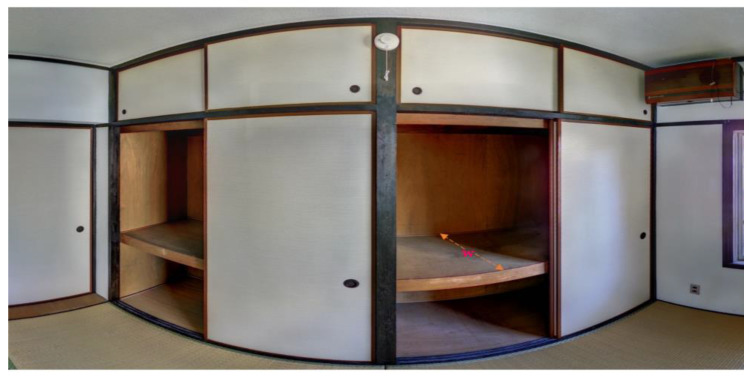
The wardrobe falsely detected as a wall.

The existing building contains a substantial number of non-load-bearing walls, whereby most of the interior walls are made up of *shôji* (traditional Japanese sliding walls or doors) and glass partitions. Since the light partitions have the same heights (2.50–3.20 m) as those of load-bearing walls (solid walls), we relied mainly on the differences in their thicknesses to distinguish the load-bearing from the non-load-bearing walls. A thickness tolerance (*t_w_*) applied in the classification (for 10 cm ≤ *t_w_* ≥ 50 cm) managed to dismiss most of the light partitions except for a few partitions with thickness >10 cm, which accounted for the FN. The value of the FN was also attributed to the incorrect classification of certain wardrobes, which displayed dimensions (height and width) that covered the full extent of one face of a wall and a breadth (w) not exceeding 50 cm as shown in Figure 23. The FP value was contributed to mostly by the undetected external walls. This was due to the double layer façades, which restricted the scanner’s access in the narrow spaces leading to the limited exposure of the exterior faces of the external walls. The presence of trees in the vicinity of the building also partly limited the detection of the external walls.

### 5.2. Evaluation of Plane Extraction and Segmentation

Figure 14b shows an input (subsampled) point cloud *P* with a total of 30,129,150 points, and after the spatial decomposition, the planar patches are derived and classified accordingly into class elements. As a result of the segmentation and classification processes, the remaining points (*₱*) are found to represent about 35% of the original points (*P*) such that *₱* ⊆ *P*.

Analysis was conducted on the remaining points (*₱*) to gain insight into the relevancy of the unclassified points. We manually inspected the remaining planar patches to determine if they represented the surfaces of the class objects. Among them, about 20% were found to be part of the class objects largely associated with the external walls, which also contributed in large part to the FP value for the walls. It was observed that they lacked appropriate pair patches to form the principal patches in accordance with the conditions set in Section 3.5.1. Almost 90% of the unclassified vertical pairs (ℿ*_v_*), represented the drywall partitions and doors, which were successfully rejected on the basis of thickness (*t_w_*).

### 5.3. Comparative Evaluation

**Figure 24:**
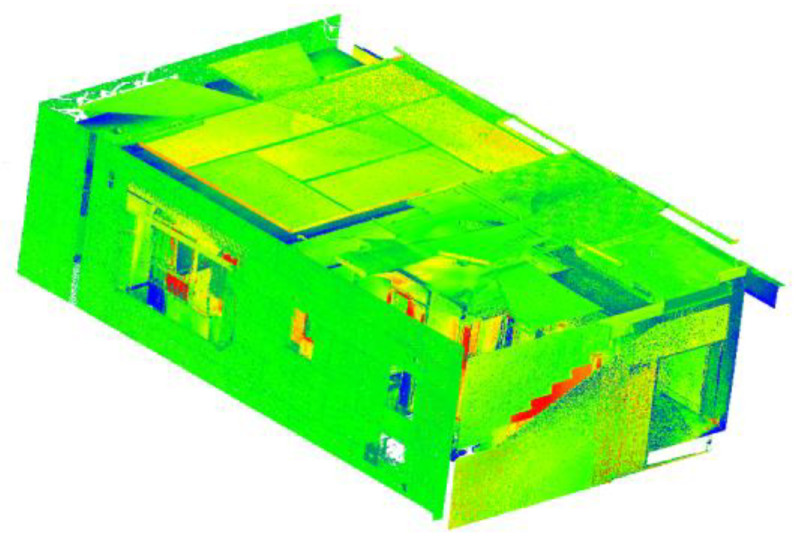
Original point cloud used for comparative evaluation.

In order to compare the performance of the proposed method with the recent outstanding clustering methods, we used a different point cloud cut from the original dataset (refer to Figure 14a) for an independent evaluation. A relatively smaller point cloud was used in this section for general comparison as shown in Figure 24. Two methods were selected for this task; the density-based clustering approach as proposed in [36], and an optimized DGCNN with the neighbor network as proposed in [17]. As for the method in [17], we used the dataset from the Stanford 3D Indoor Space (S3DIS) for training. These methods were selected because they demonstrated good segmentation results without considering the intensity and color information in their indoor applications; in particular, [17] dealt with missing points in its modeling pipeline.

**Figure 25:**
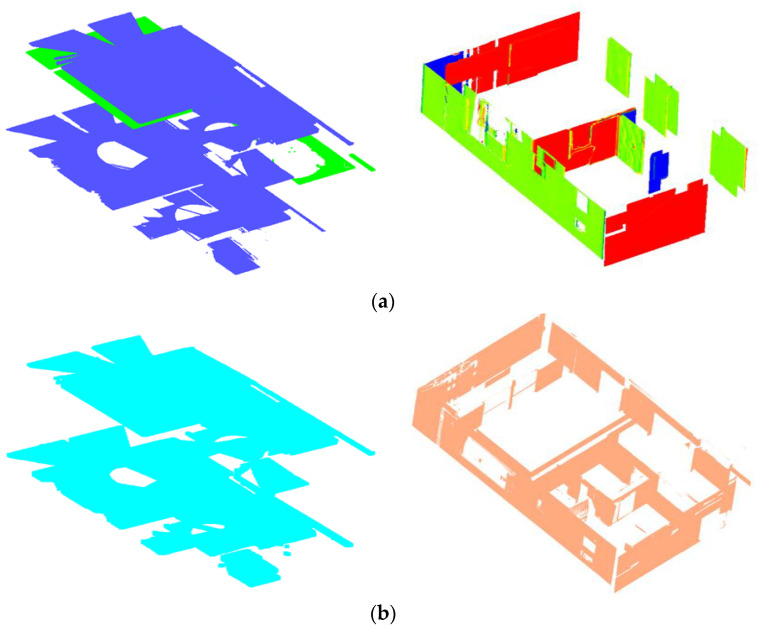

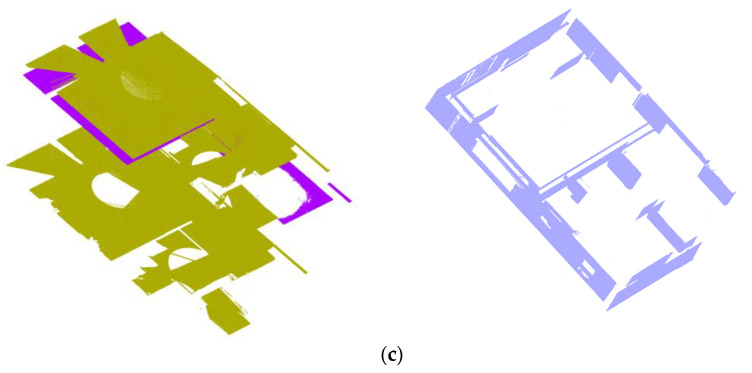
Results of segmentation of floors, ceilings, and walls: (**a**) proposed approach; (**b**) density-based clustering [36]; (**c**) DGCNN with the neighbor network [17].

From Table 3, it can be seen that all three methods generally performed well in segmenting the floor slab and wall classes as shown in Figure 25. The proposed method had an edge advantage in segmenting beams, while the density-based approach showed a weak performance in this category due to low scores in all metrics. The method in [36] is prone to a lower recall value due to overreliance on local density, which fails to distinguish objects in the vicinity as in the case of beams and ceilings.

Other methods have shown slight advantages in segmenting wall class compared to the proposed method, which is attributed to the pairwise requirement in our proposed approach. Other methods need only one planar surface to produce a candidate wall class.

### 5.4. Limitations and Future Works

This study presents a method to segment raw point clouds into meaningful classes representing generic structural elements. The approaches proposed in this study have shown the potential to classify even the abstract-oriented members including the non-horizontal and non-vertical members. However, the method is strictly focused on segmenting the planes and forgoes the opportunity to further process the points and surface patches embodied in the planes.

On that account, we are working on several improvements to modify and advance our segmentation method. Firstly, we are working on examining the missing regions found between the surface patches in order to objectively detect openings such as doorways and windows from occlusions. This is necessary for further refining the patches and knowing what gaps to refill during 3D inpainting processing for 3D remodeling [69].

Secondly, we are working on efficient ways to allocate points when patches intersect with one another. This is part of the boundary regularization process, whereby previous studies have worked on boundary accuracy on the patch margins and around the openings. However, assigning points falling on the intersection line to an appropriate surface patch will further improve the segmentation process. Thirdly, from the experimental results, we have observed that our method is not performing well in classifying the unpaired surface patches as the result of occlusions. Methods that can improve the approach towards classifying the single planes are significant especially dealing with buildings with small rooms where the presence of small objects can occlude a larger part of its walls.

Fourthly, more experiments are being prepared to examine our proposed method in various settings to assess the robustness of our thresholds applied in the study. The proposed method has only been tested on a regular rectangular building with minimal variations of floor layouts and configuration of the main components. Further tests on other building conditions are necessary to evaluate the performance of the proposed method and recognize other areas of improvement.

Also, we are looking for ways to incorporate deep learning into the knowledge-based framework proposed in the study. Deep learning will provide an opportunity to enhance the automatic learning of points and give more insights into the segmentation process.

Finally, since the main goal of the segmentation process is to perform thorough LCA, which involves calculations of the material quantities incorporated in the existing building, we are dedicated to producing volumetric modeling of the segmented points. Our current method only produces surface modeling in plane pairs; hence, transforming it into typical volumetric modeling is useful in reverse engineering processes, for instance, a scan-to-BIM [13,70].

## 6. Conclusions

This research is based on the classification of point clouds into structural components based on the attributes of the extracted planar patches and their spatial dependencies. The developed method is composed of three main parts. Based on the input raw point cloud characterized by high clutter and noise, the first part deals with extracting minimal planar patches from the voxel units, and empirically merging together the adjoining patches sharing similar saliency features. The second part consists of merging the disconnected coplanar patches, which involves determining the pairs of principle patches and using them as proxies to draw out distant coplanar patches. The process continues with spanning the best-fit planes for the principal patches and empirically assigning the coplanar patches and points that are found within a signed point-to-plane distance followed up by a prudent vetting process to eliminate outliers.

The third part of the process involves segmenting the planes and appropriately classifying them into class elements which are: floor slabs, floor beams, walls, and columns. Each class element is performed individually starting with detecting the floor slabs, then the floor beams, and finally classifying the walls and columns together.

The approach experimented on a large point cloud of an existing building that contained a high ratio of occlusions and clutter. The entire segmentation process was applied using the same proposed thresholds and other defined criteria as explained in Section 2. The proposed method was evaluated for its qualitative and quantitative performance. The test results generally produced positive results, with each element being satisfactorily classified. The floor slabs were 100% detected and classified. The constituent surfaces composing the floor slabs (ceilings and floors) were also perfectly identified. The floor beams showed a promising performance with a precision of 62% and the ability to positively recall 88% of beams. Good results were also observed in detecting the walls, where the approach was performed at a precision level of 65% and with the ability to recall 83% of walls, while the worst-case scenario was observed at 58% only. Most of the class elements were appropriately labeled. We also compared our proposed method with other recent and reputable segmentation methods for building components and the performance results in wall and floor classes were fairly balanced, while our proposed method showed more advantage in segmenting beams.

The general performance of the method was highly affected by the insufficient pairing of planes largely due to occlusions on one side of the pair-set. Hence, while the method showed good potential to appropriately segment point clouds into class elements, especially the horizontal members, further research is necessary to enhance its performance in segmenting wall classes. Also, this research is mainly described using planar building components; however, due to variants in the structural forms of buildings, there is a need to adopt other geometrical representations in the approach.

## Figures and Tables

**Table 1:** The number of scan stations, the total number of points, and registration precision.

Experiment	No. of Scan Stations	No. of Points	Average Resolution Range	Average Range Error
Original Point cloud	236	374,405,029	0.1 mm	0.25 mm rms
Test-data	N/A	66,997,720	0.1 mm	0.25 mm rms

**Table 2:** Quantitative evaluation of the classification of points into class elements.

Metrics	Floor Slabs			Floor Beams			Walls
	Ceilings	Floors	^1^ Slabs	Soffits	Vertical Sides	^1^ Beams	
TP	4	5	5	192.00	149.76	149.76	202.00
FP	0	0	0	92.16	92.16	92.16	108.00
FN	0	0	0	20.80	20.80	20.80	40.00
TN	0	8	8	23.40	23.40	23.40	28.00
Precision	1	1	1	0.68	0.62	0.62	0.65
Recall	1	1	1	0.90	0.88	0.88	0.83
F1_-score_	1	1	1	0.77	0.73	0.73	0.73
IoU	1	1	1	0.63	0.57	0.57	0.58

^1^ Slabs and ^1^ Beams are subject to the identification of all their constituent parts.

**Table 3:** Comparison among different segmentation methods.

Objects	Floor Slabs			Floor Beams			Walls		
* Metrics	P	R	F1	P	R	F1	P	R	F1
Proposed method	1	1	1	0.75	0.75	0.75	0.60	0.75	0.67
Density-based clustering [36]	1	1	1	0.25	0.33	0.29	0.70	0.64	0.67
DGCNN with the neighbor network [17]	1	1	1	0.67	0.67	0.67	0.70	0.70	0.70

* P—Precision, R—Recall, and F1—F1_-score_.
